# Association of adenylate cyclase activity in vasopressor-type neurally mediated syncope based on the *α2b-AR* gene

**DOI:** 10.1371/journal.pone.0317817

**Published:** 2025-02-03

**Authors:** Tomoyoshi Komiyama, Kengo Ayabe, Kunzo Orita, Ayumi Sasaki, Yuka Kitamura, Hideyuki Matsuzawa, Teppei Yamashita, Eiichiro Nagata, Yasuhiro Kanatani, Koichiro Yoshioka

**Affiliations:** 1 Department of Clinical Pharmacology, Tokai University School of Medicine, Isehara, Kanagawa, Japan; 2 Cardiovascular Center, Miyazaki Medical Association Hospital, Miyazaki, Miyazaki, Japan; 3 Department of Cardiology, Tokai University School of Medicine, Isehara, Kanagawa, Japan; 4 Okayama University Medical School, Okayama, Okayama, Japan; 5 Department of Life Science Support, Research Innovation Center, University Hospitals Sector, Tokai University, Isehara, Kanagawa, Japan; 6 Department of Neurology, Tokai University School of Medicine, Isehara, Kanagawa, Japan; Cinvestav-IPN, MEXICO

## Abstract

Neurally mediated syncope (NMS) arises from a neural reflex; however, its underlying cause remains unknown. Previous research has shown that variations in the Gi-α signal transduction rate led to changes in adenylate cyclase (AC) activity levels. Thus, we hypothesized that these fluctuations in AC activity could contribute to NMS. This study aimed to investigate the receptor genes associated with glutamate (Glu) repeat polymorphism sites Glu12 and Glu9 in the *α2B-AR* gene. A total of 50 patients with vasodepressor-type (VT)-NMS and 20 healthy volunteers were included in this study. We assessed AC activity levels and blood pressure responses during the head-up tilt (HUT) test and conducted a Glu repeat polymorphism analysis to explore its potential association with NMS. Our findings showed significantly higher AC activity in patients with the Glu12/12 homotype than healthy volunteers across all four HUT test points. Conversely, patients with the Glu9/12 heterotype exhibited a significant difference only 10 min after the test initiation, suggesting a pronounced activation effect of the *β2-AR* Gs-α subunit in these individuals. Both genotypes displayed the greatest blood pressure fluctuations under 70° tilt stress, with patients with Glu12/12 showing consistent cardiac load and higher values than those with Glu9/12 at two points. Notably, the frequency of NMS onset within 20 min during the tilt test varied, with one Glu12/12 patient and seven Glu9/12 patients experiencing syncope. Additionally, patients with the Glu9/12 heterotype were found to have a higher risk of syncope after prolonged standing compared to those with the Glu12/12 homotype. These results suggest that patients with the Glu12/12 homotype exhibit elevated AC activity levels, which may help increase blood pressure to prevent syncope. This study underscores that variations in AC activity among different gene types could influence the frequency of NMS onset during standing.

## Introduction

Syncope, characterized by a temporary loss of consciousness triggered by nerve reflexes, manifests through symptoms such as bradycardia (slow pulse) and a decrease in blood pressure resulting from peripheral blood vessel dilation [1, 2]. Neurally mediated syncope (NMS), a variant of syncope, occurs frequently and significantly impacts activities of daily living [3–5]. Despite patients with NMS generally having a favorable prognosis, the underlying mechanism and optimal treatment strategies remain unclear [6, 7]. As a result, since 2011, our research team has been dedicated to investigating syncope, focusing on unraveling the mechanisms underlying NMS. Specifically, we have explored potential causes of NMS through various tests, including assessments of plasma adrenergic concentrations, electrocardiograms, blood pressure measurements, α2B-AR adrenergic polymorphism analysis, and adenylate cyclase (AC) activity evaluations during resting and head-up tilt (HUT) tests [8–11]. Our findings revealed notable differences in AC activity levels between patients with NMS and healthy volunteers both at rest and during the HUT test [10, 11]. Additionally, we found that there is a possibility of distinguishing between vasodepressor type (VT) or mixed type syncope by examining AC activity levels [11]. Moreover, our results highlighted significant differences in blood pressure and AC activity levels during the HUT test between patients with VT-NMS and healthy volunteers [10], and an increase in active AC corresponded to a decrease in blood pressure of approximately 10 min in these patients. In VT-NMS cases, the rise in AC activity preceding syncope was associated with a subsequent drop in blood pressure [10], i.e., the dilation of blood vessels and subsequent syncope in patients with VT-NMS appeared to result from increased AC activity. Conversely, it can also be inferred that patients with VT-NMS are predisposed to fainting due to high blood pressure and AC activity at rest [10]. Consequently, these findings may serve as indicators for diagnosis and treatment in facilities where tilt testing is not feasible. Thus, providing lifestyle guidance to patients with suspected NMS, such as advising against prolonged standing for more than 10 min, may aid in syncope prevention.

This study aimed to investigate the correlation between genetic mutations serving as markers and AC activity to enhance the precision of NMS diagnostic research. Previously, we hypothesized that the suppression duration of AC activity would be prolonged during NMS [8]. However, our earlier results suggested differences in the predicted binding energies of *α2B-AR* gene and Gi-α protein-coupled receptors (GPCRs) between Glu9 and Glu12 repeats, with the Glu9/12 heterotype potentially exerting varied effects on vasoconstriction signals [8]. Furthermore, the binding energy of the Gi α-subunit in Glu9 was stronger than in Glu12, implying that the receptor containing the Glu12 repeat might rapidly dissociate from the β and γ subunits, thereby influencing AC signaling [8].

This study examined the relationship between adrenergic polymorphism (α2B-AR) and AC activity, exploring their potential link to NMS development. Small et al. [12] had previously reported differences in AC activity between Glu12/12 and Glu9/9 repeats in experiments utilizing hamster ovary cells, suggesting potential similarities in human AC activity effects. Additionally, we elucidated the connection between the number of Glu repeats and AC activity levels, along with how variations in the number of Glu repeats influence NMS onset. Polymorphisms in adrenergic receptor genes are associated with the pathology and hemodynamics of heart failure in humans, with studies indicating links to diseases such as heart failure, diabetes, obesity, and polycystic ovary syndrome in Asian, European, African, and South American populations [8, 13–17]. However, no prior studies have reported the relationship between NMS and AR polymorphisms of adrenergic receptor genes. Therefore, this study aimed to clarify the interaction between guanine nucleotide-binding protein (Gi) and AC through in silico analysis. AC plays a pivotal role in mediating the effects of Gi protein on blood vessel contraction and relaxation via adrenergic receptor α1 or β2 [11–13]. For instance, activating the adrenergic subtype β2 receptor enhances AC binding to Gs protein, leading to cyclic adenosine monophosphate (cAMP) production [13, 18–20]. Subsequent activation of protein kinase A by cAMP facilitates calcium ion channel opening, thereby accelerating calcium uptake by the sarcoplasmic reticulum [21–27]. Consequently, increased calcium concentration correlates with enhanced smooth muscle contractile force [21–24, 28–30].

**Fig 1:**
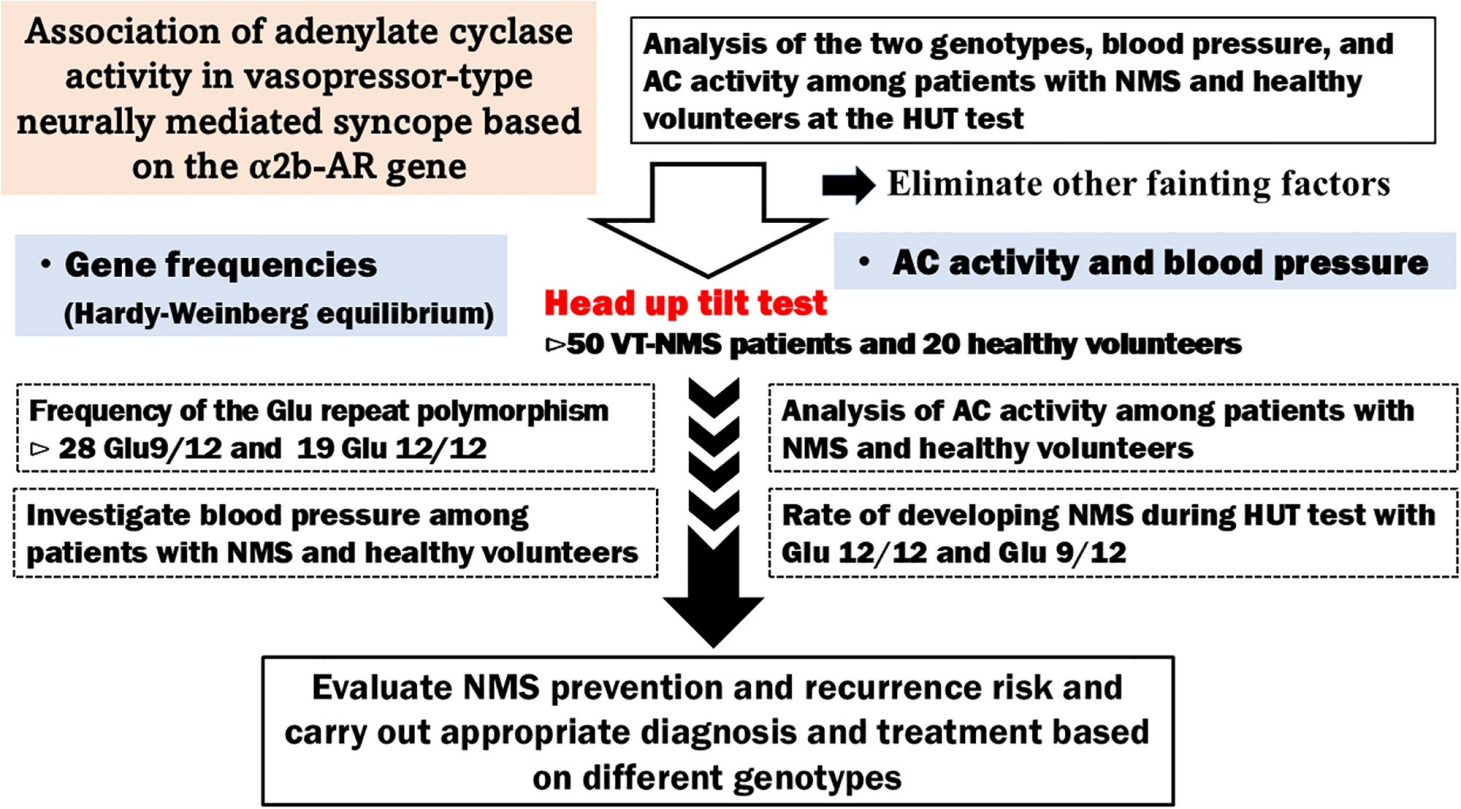
Overview of this study.

Hence, we propose a novel study based on the heterogeneous polymorphisms of healthy volunteers and patients with NMS, alongside clinical trial results (HUT and AC activity tests). Given that past clinical studies have not provided evidence linking *α2B-AR* gene polymorphisms with AC activity levels in patients with NMS, we aim to propose effective treatments and preventive measures against cardiovascular diseases during acute stress through this research, as this will be useful for the diagnosis of NMS (Fig 1).

## Materials and methods

### Ethics statement

This study was reviewed and approved by each participating university’s Institutional Ethics Committees (approval number: 14R-053). Prior to participation, all patients provided written consent for both clinical research and genetic studies, following the approval of the experimental procedure by the relevant ethics committee at Tokai University.

The study period is July 30^th^ 2014-March 31st 2025. Informed consent was obtained by explaining the procedure verbally and in writing to patients and their families over 1–2 hours. In the case of minors under the age of 20, consent was obtained verbally and in writing with the presence of a guardian. Also, the last date of access to the data was April 8th 2024 and authors did not have access to individual participants information.

### Patients with NMS and healthy volunteers

This study comprised 50 patients with NMS and 20 healthy volunteers (Table 1 and S1 Table), all recruited from the Tokai University School of Medicine between June 2016 to March 2023 [11]. All patients underwent examination and treatment in accordance with the Japanese Circulation Society (JCS) guidelines (http://j-circ.or.jp/english/), which aligned with the 2009 European Society of Cardiology guidelines [31]. Furthermore, none of the patients in this study had a history of brain or heart diseases or disorders.

**Table 1:** Genotype proportion and age in patients with NMS and healthy volunteers.

Genotype	Patients with VT-NMS (n = 50)	Healthy volunteers (n = 20)
**Glu12/12**	n = 19	n = 12
Age (years) mean	39.7±19.3	35.8±9.2
**Glu9/12**	n = 28	n = 7
Age (years) mean	32.2±18.7	37.7±8.6
**Glu9/9**	n = 3	n = 1
Age (years) mean	48.7±12.3	29.0

### DNA extraction, polymerase chain reaction amplification, sequencing, and cloning of Glu repeat regions

DNA extraction was performed from the peripheral blood mononuclear leukocytes. The leukocytes were treated with high molecular weight (HMW) Buffer and incubated at 60°C for 30 min [8]. Subsequently, 10 μL of proteinase K was added, and the leukocyte solution was further incubated at 60°C. DNA extraction was carried out using neutralized phenol/chloroform, followed by ethanol precipitation. The resulting solution was dissolved in TE buffer, and the DNA concentration was determined by measuring the absorbance at 260 nm (A260), adjusted to 10 ng/μL. Four primers targeting the α2B-AR gene (1,353 bp) were designed, and PCR was conducted using KOD FX neo (Tables 2 and 3). These primers were designed to amplify a fragment of the *α2B-AR* gene based on the human adrenergic *α2B-AR* receptor gene sequence from GenBank at the National Center for Biotechnology Information (NCBI). PCR amplifications involved heat denaturation at 98°C for 20 s, followed by 35 cycles of heat denaturation at 98°C for 5 s, and annealing/extension at 68°C for 40 s. Four microliters of the amplified PCR product underwent electrophoresis (1% Agarose, TBE 50 V, 60 min) to ensure sufficient product amplification and the absence of minor bands. Following this, 1 μL of EXOSAP-IT was added to 6 μL of the remaining PCR product, and the mixture was incubated at 37°C for 30 min, followed by 80°C for 15 min. After EXOSAP-IT treatment, 3 ρmol primer was added to 1 μL of the PCR product, which was then labeled with ABI PRISM BigDye Terminator v3.1 Cycle Sequencing Kits, and DNA sequencing was performed using ABI PRISM 3500xL. Subsequently, sequences were assembled and aligned using MEGA X, and the number of repeats was confirmed [32]. The 70 nucleotide sequences of α2B-AR, one from each of the 70 samples, were aligned using CLUSTALW [8]. We verified that Glu repeats constituted polymorphism sites at the Glu 298–309 position, with repeat numbers of 9 or 12 (Table 2). These alignments utilized ABY87536.1 for Glu 12 and AAK01635.1 for Glu 9 from the NCBI database [8].

**Table 2:** Number of glutamic acid Glu (E) repeats found in the α2B-AR gene region.

**Same site**	*	*	*	*	*	*	*	*	*	*	–	–	–	*
**Glu 12**	A	E	E	E	E	E	E	E	E	E	E	E	E	C
**Glu 9**	A	E	E	E	E	E	E	E	E	E	–	–	–	C
**Site number of amino acid**	297	298	299	300	301	302	303	304	305	306	307	308	309	310

In humans, two forms of Glu have been identified: homotype (9/9 and 12/12 repeats) and heterotype forms (9/12 repeats).

A: alanine; C: cysteine; D: aspartic acid; E: glutamic acid; P: proline; Q: glutamine.

*Same site in the Glu12 and Glu9 repeats.

**Table 3:** The four primer sets for theα2B-AR gene.

1	α2B-AR_1F	Forward	5′-GGGCGACGCTCTTGTCTA-3′
2	α2B-AR_1R	Reverse	5′-ACTTCGAGTGTCCGTTGACC-3′
3	α2B-AR_2F	Forward	5′-ATCTACCTGATCGCCAAACG-3′
4	α2B-AR_2R	Reverse	5′-ATGAGGCCTACAGGATCTGG-3′

### Head-up tilt test and blood collection

**Fig 2:**
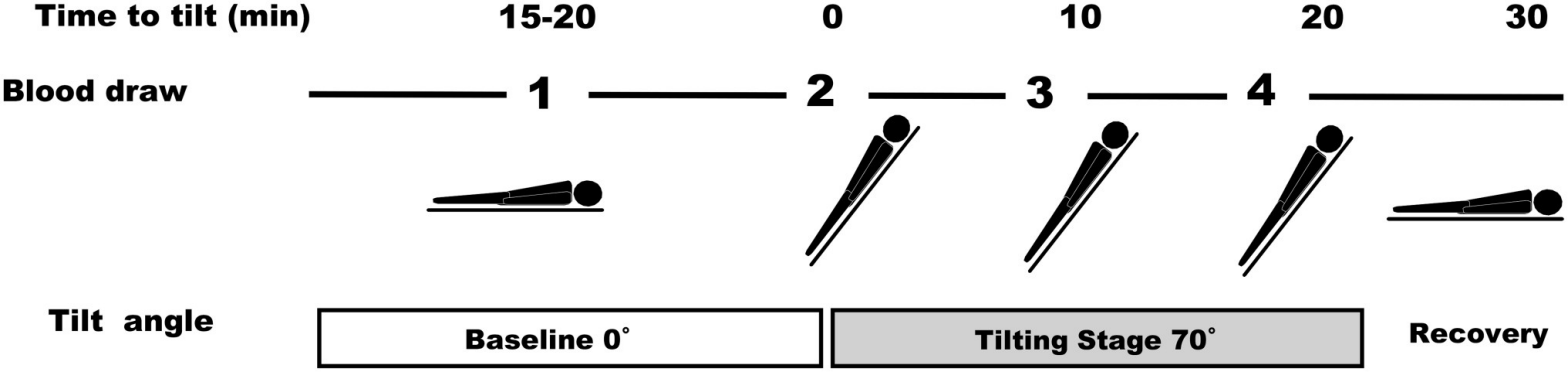
The head-up tilt table test and the four designated blood collection points. Blood samples were collected four times during the head-up tilt table test: at baseline, at 70° tilt, after 10 min, and after 20 min [11].

After obtaining informed consent, all patients were admitted to the hospital overnight [9–11]. The following day, the patients, who were neither sedated nor allowed access to food, underwent HUT table testing between 9:00 am and 11:00 am. The tilt table was set to 70°, with patients resting in the supine position for 10–20 min before baseline measurements were recorded. Similarly, healthy volunteers also underwent HUT table testing while fasting. The lights and air conditioners were turned off during the tests to minimize environmental stimuli. Blood samples (8 ml) were collected four times during the HUT table test: at baseline, at 70° tilt, after 10 min, and after 20 min (Fig 2). Simultaneously, blood pressure and pulse were measured during each blood sample collection. Venous blood (8 ml) was collected from the forearm vein using an indwelling butterfly needle, and the needle was heparin-locked. Approximately 1 ml of blood was removed initially to clear the heparin in the tube before collecting the 8 ml sample. Pharmacological provocation was avoided to prevent false-positive responses, and patients were told to refrain from consuming caffeine or medication a day before the test. A positive outcome was defined as the occurrence of syncope or presyncope accompanied by significant arterial hypotension. Blood pressure, pulse rate, and electrocardiogram were monitored throughout the HUT table test.

### Measurement of adenylate cyclase activity

#### Preparation of lymphocytes

To examine the lymphocytes, we isolated the lymphocyte layer from the blood via centrifugation using Vacutainer Blood Collection Tubes (BD, New Jersey, NY, USA). Next, the lymphocytes were washed with an induction buffer (RPMI 1640; Thermo Fisher Scientific, Inc., Waltham, MA, USA) to separate the platelets and isolate lymphocytes. The isolated lymphocytes were then treated with the test reagent (10 μM for 10,000 cells) for 30 min at 25°C. The measurement of cyclic adenosine monophosphate (cAMP) was conducted using the Promega cAMP-Glo Assay protocol (GloMax^®^-Multi Detection System, Wisconsin, USA), [10, 11] with values confirmed using a standard curve [11]. The concentration of adrenaline (AD) combined with lymphocytes was examined at 10 μM. AC activities were assessed in the presence of induction buffer and/or test reagents (basal) and 100 μM forskolin (FK). In this study, FK concentrations reached a plateau at 100 μM, and AD activity levels were expressed as percentages of FK-stimulated activity [8, 11, 12, 33, 34]:

The calculation of AC activity level was as follows:

ACactivitylevel=amountofcAMPproducedbyAD/amountofcAMPproducedat100μMFK

[11].

#### Analysis of the frequency of the α2B-AR p. Glu 298–309 polymorphism

The allele frequencies of Glu12 (*p*) and Glu9 (*q*) were determined using allele counting (Table 2). Analysis of potential deviations in genotype distribution from those expected for a population under the Hardy–Weinberg equilibrium was conducted using Pearson’s chi-squared test [8, 35]. In this test, the frequencies of Glu12 and Glu9 were denoted as p and q, respectively.

Hardy-Weinberg equilibrium: (*p* + *q*)^2^ = 1

Pearson’s chi-squared test (*χ*^2^): $\sum \frac{{\left(O-E\right)}^{2}}{E}$

Where *E* = Expected value and *O* = Observation

### Statistical analysis

All data were presented as mean ± standard deviation (SD). Comparisons were performed using *t*-tests, as appropriate. The analysis was performed using Microsoft^**®**^ Excel 365 MSO (version 2403 Build 16.0.17425.20176). The Excel *t*-test and mean ± SD program were utilized to ascertain significant differences [11]. Statistical significance was set at *p* < 0.05. Excel was also employed to analyze the ratio of AC activity.

## Results

### Frequency of the Glu repeat polymorphism

Of the 50 patients with NMS, 19 had the 12/12 homotype, 28 had the 9/12 heterotype, and 3 had the 9/9 homotype. Among the 20 healthy volunteers, there were 12 individuals with the 12/12 homotype, 7 with the 9/12 heterotype, and 1 with the 9/9 homotype. Table 1 presents the frequencies of Glu12/12, 9/12, and 9/9 repeats among 50 patients with NMS and 20 healthy individuals of Japanese descent.

Three genotypes were identified with the following allele frequencies: 12/12 homotype—19 (0.37) patients; 12/9 heterotype—29 (0.57) patients; and 9/9 homotype—3 (0.06) patients (Table 4). Among the 20 healthy volunteers, the allele frequencies were as follows: 12/12–12 volunteers (0.60), 9/12–7 volunteers (0.35), and 9/9–1 volunteer (0.05). Additionally, the expected frequencies from the Hardy–Weinberg equilibrium were suggested to be 0.4356 (12/12), 0.4488 (9/12), and 0.1156 (9/9).

**Table 4:** Allele frequency of three Glu repeat types.

Genotype	N	Observed frequency	Expected frequency from Hardy–Weinberg
**Glu 12/12**	19	0.38	0.4356
**Glu 9/12**	28	0.56	0.4488
**Glu 9/9**	3	0.06	0.1156
**Total**	50	1.0	1.0

The null hypothesis posits that the population is in Hardy–Weinberg equilibrium, while the alternative hypothesis suggests that the total ethnic population is not in equilibrium. With one degree of freedom, the significance level for one degree of freedom at 5% was calculated to be 3.84. The null hypothesis was accepted since the χ^2^ value was <3.84, indicating no observed bias in the healthy volunteers.

### Analysis of AC activity among patients with NMS and healthy volunteers at the HUT test

AC activity was investigated in 50 patients with NMS and 20 healthy volunteers (Table 5 and S2 Table). The results of the HUT test indicated the following p-values: at baseline, *p* = 0.006; at 70° tilt, *p* = 0.007; at 10 min, *p* = 0.017; and at 20 min, *p* = 0.09. Patients with VT-NMS showed significantly higher values at three-time points. It is also worth noting that one of the 50 patients with VT-NMS had an error in the AC activity data and was consequently excluded from our analysis.

**Table 5:** Analysis of AC activities among patients with NMS and healthy volunteers at the head-up tilt test.

HUT test	VT-NMS n = 50	Volunteers n = 19	*p*-value
**Baseline**	0.66±0.19	0.56±0.13	0.006
**70° tilt**	0.63±0.20	0.53±0.13	0.007
**10 min**	0.67±0.22	0.56±0.18	0.017
**20 min**	0.64±0.22	0.57±0.16	0.09

### Analysis of the two genotypes, blood pressure, and AC activity among patients with NMS and healthy volunteers at the HUT test

**Fig 3:**
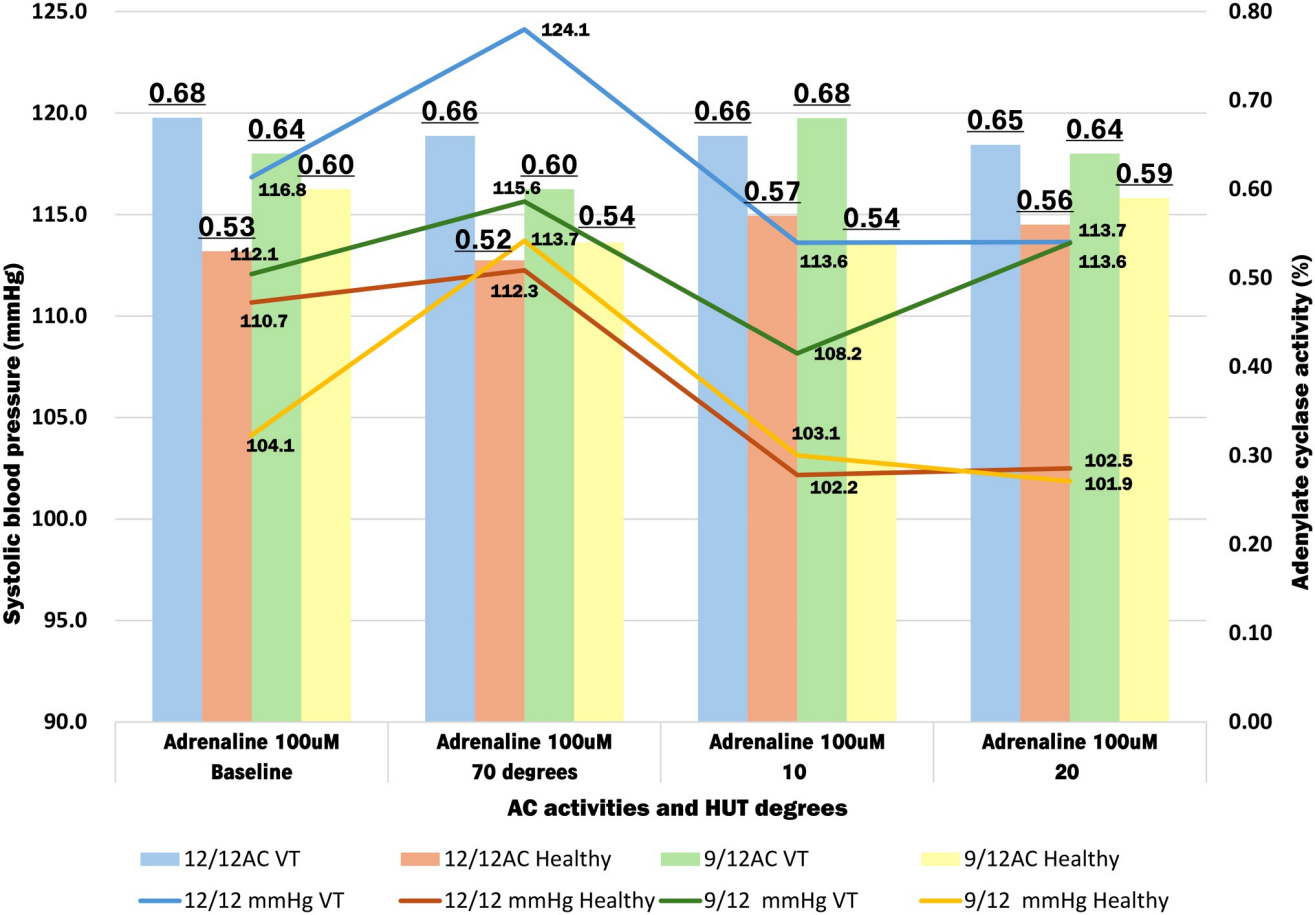
The relative amount of AC activity and systolic blood pressure by differences in the number of Glu repeats during HUT.

We measured the AC activity for the two genotypes (Glu12/12 and Glu9/12) among the 50 patients with NMS and 19 healthy volunteers (Fig 3, Table 6 and S3 Table). Among the 19 patients with NMS with Glu12/12, the AC activity was significantly higher than those of 12 healthy volunteers at baseline (*p* = 0.007) and 70° tilt (*p* = 0.01) during the HUT test. Similarly, among the 28 NMS patients with the Glu9/12 heterotype, a significantly higher value (*p* = 0.03) was observed after only 10 min of stress.

**Table 6:** AC activity changes during HUT by the genotype of Glu12/12 and Glu9/12.

HUT test	Glu 12/12			Glu 9/12		
	VT-NMS n = 19	Volunteer n = 12	*p*-value	VT-NMS n = 28	Volunteer n = 7	*p*-value
**Baseline**	0.68±0.20	0.53±0.11	0.007	0.64±0.18	0.60±0.17	0.31
**70 degrees**	0.66±0.20	0.52±0.13	0.01	0.60±0.17	0.54±0.14	0.17
**10 min**	0.66±0.22	0.57±0.22	0.13	0.68±0.21	0.54±0.13	0.03
**20 min**	0.65±0.24	0.56±0.19	0.12	0.64±0.19	0.59±0.13	0.22

This data used adrenaline 100 μM.

Furthermore, we observed that the AC activity was higher in the Glu12/12 homotype compared to the Glu 9/12 heterotype (Table 6). However, no significant differences were found in AC activity at the four tilt points between patients with Glu12/12 NMS and those with Glu9/12 NMS (S3 Table).

Tables 6 and 7 demonstrate notable observations regarding AC activity and systolic blood pressure variations among different genotypes during the HUT test (S3 and S4 Tables). For patients with Glu12/12 experiencing NMS, AC activity remained consistent across all four-time points, ranging between 0.68 and 0.69. In contrast, patients with Glu9/12 experiencing NMS exhibited fluctuations in AC activity, with the lowest recorded at 0.57 during 70° tilt stress and the highest at 0.64 at the 10-minute mark. A significant difference was observed between 70° tilt and 10-minute time points, indicating considerable variability in the patients with Glu9/12 experiencing NMS. The Glu 9/12 levels in healthy volunteers exhibited minor fluctuations, whereas significant fluctuations were not observed in patients with NMS. Blood pressure values were elevated in Glu12/12 and Glu9/12 patients with NMS during 70° tilt stress compared to healthy volunteers. Specifically, Glu 12/12 (NMS) showed significant differences from Glu 9/12 (healthy volunteers) at baseline, at 10 min and 20 min (*t*-test 1, Table 7 and S4 Table). Glu 9/12 (patients with NMS) also displayed significant differences compared to Glu9/12 (healthy volunteers) at baseline and 20 min (*t*-test 2, Table 7 and S4 Table). Additionally, Glu12/12 (patients with NMS) exhibited significant differences compared to Glu12/12 (healthy volunteer) at 70° tilt, 10 min, and 20 min (*t*-test 3, Table 7 and S4 Table). Furthermore, Glu12/12 (healthy volunteers) demonstrated a significant difference (*p* = 0.04) at 20 min (*t*-test 4; Table 7 and S4 Table). Although Glu12/12 consistently showed higher blood pressure values than Glu9/12 across all time points, the difference was not statistically significant (*t*-test 5, Table 7 and S4 Table).

**Table 7:** Changes in blood pressure (systolic) during HUT for Glu12/12, Glu9/12, and healthy volunteers.

		Baseline	70° tilt	10 min	20 min
**Average**	9/12 NMS (n = 28)	112.1±15.1	115.6±19.4	108.2±23.3	112.2±20.0
**Average**	12/12 NMS (n = 19)	116.8±13.9	124.1±16.2	113.6±14.0	112.4±16.1
**Average**	9/12 HV (n = 7)	104.1±8.6	113.7±16.1	103.1±10.8	101.9±10.2
**Average**	12/12 HV (n = 12)	110.7±9.2	112.3±16.6	102.2±12.6	102.5±11.0
***t*-test 1** *p*-value	HV9/12 vs. NMS12/12	0.006 (diastolic: 0.005)	0.09	0.03	0.03
***t*-test 2** *p*-value	HV9/12 vs. NMS9/12	0.04 (diastolic: 0.04) (plus: 0.04)	0.40 (plus: 0.002)	0.21 (plus: 0.003)	0.04 (plus: 0.003)
***t*-test 3** *p*-value	HV12/12 vs. NMS12/12	0.07	0.03	0.01	0.03
***t*-test 4** *p*-value	HV12/12 vs. NMS9/12	0.36	0.29 (plus: 0.01)	0.16 (plus: 0.01)	0.04 (plus: 0.02)
***t*-test 5** *p*-value	NMS9/12 vs. NMS12/12	0.14	0.06	0.17	0.49

HV: healthy volunteers

Lastly, a significant difference in heart rate (t-test 2 and t-test 4) was confirmed at all three points after loading for NMS9/12 patients.

### Rate of developing NMS during HUT test with Glu 12/12 and Glu 9/12

Among the 18 Glu 12/12 type patients, only one patient (0.05%) developed NMS syncope, whereas it occurred in seven of the Glu 9/12 heterotypes (25%) (Table 8). Therefore, we inferred that the syncope rate was twice as high in the Glu 9/12 group than in the Glu 12/12 patients. All incidents of syncope occurred at the 10-minute mark.

**Table 8:** Rate of developing NMS during 20 minutes in the tilt test.

	VT-NMS number of patients	Number and percentage of patients with symptoms during tilt	Onset time after stress (min)
**Glu 12/12**	19	1 (0.05%)	12.0
**Glu 9/12**	28	7 (25.0%)	12.9 ± 4.8

## Discussion

While the research findings concerning the correlation between AC activity levels, clinical physiological tests, and *α2B-AR* gene polymorphisms in the context of NMS are considered to be highly innovative [8], we aimed to propose effective preventive measures and future treatment strategies for cardiovascular disease triggered by acute stress while also shedding light on the underlying causes of NMS through this study. Patients experiencing syncope episodes while standing or sitting are often suspected of having NMS. However, since various conditions can lead to syncope (such as orthostatic hypotension, cardiogenic causes, arrhythmia, epilepsy, and cerebral vascular disorders) [36–43], a comprehensive evaluation is necessary to isolate syncope-related symptoms. Interestingly, research has shown that in cases where NMS is suspected, a definitive diagnosis can be made through the HUT test [9].

To enhance the accuracy of the NMS diagnosis, blood samples were collected at four different time points during the tilt test. Subsequently, we investigated the frequencies of 12/12 and 9/12 *α2B-ADR* gene variants and examined the differences in AC activity and blood pressure levels between 12/12 and 9/12 genotypes. The frequencies of the *α2B-ADR* genotypes 12/12 and 9/12 associated with AC activity were analyzed in both patients and volunteers. However, mutations within this repeat region significantly affect the regulation (*α2B-ADR*) and stimulation (*β2-ADR*) of AC activity, leading to variations in the rate of signaling of AC activity [8]. In particular, our analysis revealed that this repeat region plays a role in the binding energy of the Gi-α subunit. Consequently, we posit that the differences in the binding energies of the 9 and 12 repeats impact AC activity and are implicated in the onset of NMS.

Furthermore, the frequencies of the 12/12 and 9/12 types showed no variation in NMS development, and the patient cohort utilized adhered to Hardy–Weinberg equilibrium (χ^2^ value was < 3.84) [8, 35], indicating no bias in gene frequency within this group compared to the broader Japanese population (Table 4).

Additionally, no major disparities were observed in genotype frequencies compared to our prior findings. Thus, these outcomes suggest that genotype frequencies of the 9/12 and 12/12 types persist in equivalent proportions among healthy volunteers and those with NMS, implying that gene frequency may not directly correlate with NMS onset. However, when combined with alterations in AC activity, it could facilitate a more accurate assessment of NMS risk.

Furthermore, we evaluated and compared AC activity between the two genotypes (Glu12/12 and Glu9/12) during HUT testing in 50 patients with VT-NMS and 19 healthy volunteers (Fig 3). Among the 19 patients with NMS with Glu12/12, baseline values were *p* = 0.007, and at 70° tilt, *p* = 0.01. These values were significantly higher than those of healthy volunteers at both time points (Table 5). Interestingly, significantly higher values were observed among the 28 patients with NMS with Glu9/12 heterotype, (*p* = 0.03) after 10 min of stress (Table 6). This suggests a relatively robust effect of the Gs-α subunit of the ADRβis in patients with VT-NMS (9/12 and 12/12). However, no notable differences were observed between the two genotypes. Our previous study highlighted that the Glu12/9 heterotype prolonged the inhibition of AC action compared to the Glu12/12 homotype, as evidenced by Gi-α subunit dissociation time measurement. Thus, it is plausible that the AC activity levels of the Glu9/12 heterotype in patients are suppressed, as evident by the significantly elevated values after only 10 min of stress (*p* = 0.03), with no notable differences from healthy volunteers observed at other intervals.

The changes in AC activity and blood pressure during HUT testing based on the Glu12/12 and Glu9/12 genotypes were also analyzed. AC activity remained consistent across all four HUT test points in patients with Glu12/12 VT-NMS (Fig 2). Conversely, patients with Glu9/12 VT-NMS exhibited a significant difference compared to healthy volunteers, notably evident 10 min after loading (at 70° tilt). Additionally, both Glu9/12 healthy volunteers and patients with VT-NMS showed fluctuations, with the latter experiencing pronounced variations (Fig 2). Regarding blood pressure fluctuations, both Glu12/12 homotype and Glu9/12 heterotype patients experiencing NMS exhibited peak values at 70-degree stress, with Glu12/12 consistently higher than Glu9/12 across all four points (S4 Table). This indicates persistent cardiac stress in patients with the Glu12/12 homotype while standing. Among the 19 patients with Glu12/12 experiencing VT syncope, only one (0.05%) developed NMS syncope, while seven (25%) with Glu9/12 heterotype suffered from NMS syncope. All syncope incidents resulted in fatalities within 10 min after loading, consistent with our previous findings [10]. Therefore, based on these observations, we posit that genotype considerations may help predict syncope occurrences.

Interestingly, we observed that patients with Glu12/12 experiencing VT-NMS are less likely to experience syncope when standing for > 10 min. In contrast, patients with the Glu9/12 heterotype face a heightened risk of syncope under similar circumstances. This suggests that NMS onsets, marked by a blood pressure drop (from 115.6 to 108.2) alongside a rapid 8% increase in AC activity (from 0.60% to 0.68%) upon standing, may vary based on Glu12/12 and Glu9/12 receptor polymorphisms in patients. Additionally, the pulse rate increased significantly (Table 7, t-test 2 and t-test 4) at all three points after loading, confirming that the heart was under stress. This is thought to be related to differences in the incidence of syncope (Table 8). Therefore, these results suggest that incorporating genetic typing into diagnoses could offer clearer guidance on the risk of orthostatic syncope.

Based on this study’s findings, we propose a novel treatment approach grounded in clinical statistical analysis of neuromodulated syncope and a comprehensive understanding of circulatory pathophysiology, incorporating genetic insights. Additionally, we believe we have contributed to the field of social medicine by providing valuable data that can be utilized in medical practice.

### Limitations

Despite its strengths, this study has some limitations that should be acknowledged. Firstly, our study sample comprised a relatively small cohort of patients with NMS (n = 50) and healthy volunteers (n = 20). Secondly, biochemical and HUT tests were conducted on each patient with syncope in a single day to identify those with NMS. This necessitates meticulousness due to stress induced by the HUT procedure on both patients and volunteers. Lastly, genotype categorization into Glu 12/12, Glu 9/12, and Glu 9/9 groups resulted in limited patient numbers available for analysis per genotype.

## Conclusions

Our study revealed distinct differences in AC activity between Glu9/12 and Glu12/12 genotypes during the HUT test. Specifically, patients with Glu12/12 showed a lower risk of syncope than patients with the Glu9/12 heterotype after prolonged periods of standing. This implies that individuals with the Glu12/12 genotype tend to possess heightened AC activity levels, which in turn elevate blood pressure to mitigate syncope occurrences. Consequently, variations in AC activity among different gene types may influence the frequency of NMS onset during standing. Drawing from these findings, we believe it may be plausible to identify healthy individuals at high risk of developing NMS.

## Supporting information

S1 Table Characteristics of the 50 VT-NMS patients and 20 healthy volunteers. (PDF)

S2 Table Raw data of adenylate cyclase activities by adrenaline (100 μM) from 50 VT-NMS patients and 20 healthy volunteers during the HUT test. (PDF)

S3 Table Raw data of the two genotypes and AC activity among VT-NMS patients and healthy volunteers during the HUT test. (PDF)

S4 Table Raw data on blood pressure during HUT for Glu12/12 and Glu9/12 VT-NMS patients and healthy volunteers. (PDF)
