# Supplementary material for: Association of adenylate cyclase activity in vasopressor-type neurally mediated syncope based on the α2b-AR gene
Source: PLoS One. 2025 Feb 3;20(2):e0317817. doi: 10.1371/journal.pone.0317817 (PMC11790091; doi:10.1371/journal.pone.0317817)
Supplement: S1 Table — (PDF) [file pone.0317817.s001.pdf]

**S1 Table. Characteristics of the 50 VT-NMS patients and 20 healthy volunteers.**

| VT-NMS | Number | Age | Sex | Systolic BPmmHg | Diastolic BPmmHg | Pulse |
|---|---|---|---|---|---|---|
| 12/12type n=19 | 1 | 70 | Female | 116 | 79 | 47 |
| | 2 | 46 | Female | 112 | 78 | 67 |
| | 3 | 55 | Male | 108 | 75 | 44 |
| | 4 | 20 | Male | 127 | 69 | 68 |
| | 5 | 42 | Male | 115 | 80 | 55 |
| | 6 | 21 | Male | 114 | 66 | 77 |
| | 7 | 68 | Male | 127 | 76 | 52 |
| | 8 | 54 | Female | 150 | 78 | 56 |
| | 9 | 57 | Male | 102 | 72 | 68 |
| | 10 | 21 | Male | 112 | 60 | 76 |
| | 11 | 21 | Female | 101 | 61 | 62 |
| | 12 | 35 | Female | 100 | 62 | 84 |
| | 13 | 32 | Female | 108 | 79 | 83 |
| | 14 | 71 | Male | 137 | 98 | 83 |
| | 15 | 24 | Female | 111 | 80 | 55 |
| | 16 | 19 | Male | 105 | 62 | 78 |
| | 17 | 16 | Male | 109 | 63 | 89 |
| | 18 | 26 | Female | 127 | 79 | 77 |
| | 19 | 56 | Female | 135 | 88 | 69 |
| 9/12type n=28 | 1 | 59 | Female | 111 | 73 | 48 |
| | 2 | 20 | Female | 142 | 60 | 95 |
| | 3 | 67 | Male | 129 | 81 | 66 |
| | 4 | 67 | Male | 90 | 58 | 56 |
| | 5 | 24 | Male | 108 | 71 | 59 |
| | 6 | 19 | Female | 121 | 64 | 89 |
| | 7 | 55 | Male | 115 | 85 | 81 |
| | 8 | 23 | Female | 112 | 70 | 56 |
| | 9 | 61 | Male | 138 | 113 | 57 |
| | 10 | 25 | Female | 112 | 76 | 66 |
| | 11 | 26 | Female | 108 | 72 | 62 |
| | 12 | 25 | Male | 132 | 78 | 71 |
| | 13 | 16 | Female | 110 | 61 | 67 |
| | 14 | 17 | Female | 119 | 78 | 60 |
| | 15 | 69 | Female | 118 | 67 | 67 |
| | 16 | 19 | Female | 118 | 79 | 101 |
| | 17 | 20 | Male | 104 | 67 | 68 |
| | 18 | 20 | Male | 115 | 55 | 60 |
| | 19 | 21 | Female | 95 | 56 | 70 |
| | 20 | 15 | Male | 75 | 42 | 73 |
| | 21 | 17 | Female | 99 | 69 | 71 |
| | 22 | 54 | Male | 110 | 76 | 63 |
| | 23 | 16 | Female | 102 | 51 | 60 |
| | 24 | 16 | Male | 110 | 61 | 80 |
| | 25 | 47 | Female | 138 | 102 | 74 |
| | 26 | 23 | Female | 104 | 69 | 92 |
| | 27 | 27 | Female | 106 | 73 | 60 |
| | 28 | 34 | Male | 94 | 67 | 60 |
| 9/9type n=3 | 1 | 59 | Male | 107 | 70 | 54 |
| | 2 | 35 | Male | 120 | 56 | 90 |
| | 3 | 52 | Male | 163 | 104 | 59 |
| **Healthy Volunteers** | **Number** | **Age** | **Sex** | **Systolic BPmmHg** | **Diastolic BPmmHg** | **Pulse** |
| 12/12type n=12 | 1 | 43 | Male | 114 | 75 | 63 |
| | 2 | 28 | Male | 117 | 76 | 66 |
| | 3 | 37 | Male | 116 | 83 | 67 |
| | 4 | 24 | Male | 110 | 60 | 55 |
| | 5 | 34 | Male | 129 | 76 | 75 |
| | 6 | 42 | Female | 108 | 64 | 71 |
| | 7 | 36 | Female | 109 | 67 | 87 |
| | 8 | 27 | Female | 93 | 60 | 78 |
| | 9 | 49 | Female | 108 | 64 | 64 |
| | 10 | 30 | Female | 107 | 56 | 71 |
| | 11 | 27 | Female | 107 | 71 | 75 |
| | 12 | 52 | Female | 114 | 78 | 76 |
| 9/12type n=7 | 1 | 47 | Female | 106 | 71 | 64 |
| | 2 | 42 | Female | 91 | 48 | 54 |
| | 3 | 40 | Female | 110 | 68 | 53 |
| | 4 | 38 | Female | 106 | 69 | 65 |
| | 5 | 45 | Female | 112 | 68 | 70 |
| | 6 | 27 | Female | 93 | 60 | 63 |
| | 7 | 25 | Male | 111 | 59 | 74 |
| 9/9type n=1 | 1 | 29 | Male | 114 | 68 | 52 |
