# Supplementary material for: Association of adenylate cyclase activity in vasopressor-type neurally mediated syncope based on the α2b-AR gene
Source: PLoS One. 2025 Feb 3;20(2):e0317817. doi: 10.1371/journal.pone.0317817 (PMC11790091; doi:10.1371/journal.pone.0317817)
Supplement: S2 Table — (PDF) [file pone.0317817.s002.pdf]

S2 Table. Raw data of adenylate cyclase activities by adrenaline (100 µM) from 50 VT-NMS patients and 20 healthy volunteers during the HUT test .

		1	2	3	4	5	6	7	8	9	10	11	12	13	14	15	16	17	18	19	20	21	22	23	24	25
	**NMS (VT) n=50**	**12_12**	**9_12**	**12_12**	**12_12**	**9_12**	**12_12**	**9_12**	**12_12**	**12_12**	**12_12**	**9_12**	**9_12**	**12_12**	**12_12**	**9_12**	**9_12**	**9_12**	**9_12**	**12_12**	**9_12**	**12_12**	**12_12**	**9_12**	**9_12**	**12_12**
		S010	S011	S015	S018	S020	S022	S025	S027	S028	S030	S033	S038	15S041	15S043	15S046	15S047	15S049	15S051	15S052	15S054	15S056	15S057	15S058	15S059	15S061
**Baseline**	Adrenaline 100uM	0.684176	0.827716	0.532215	0.572592	0.60253	0.748171	0.650868	0.567394	0.742349	0.653562	0.704688	0.431794	0.787712	0.805212	0.701491	0.645822	0.532076	0.684692	1.087747	0.613364	0.643871	0.739525	0.854632	0.902964	0.748586
**70degree**	Adrenaline 100uM	0.645164	0.773656	0.477209	0.616481	0.554981	0.742601	0.614422	0.516414	0.720865	0.518816	0.66297	0.43818	0.752473	0.849796	0.734708	0.669328	0.468116	0.619155	1.072851	0.606822	0.617435	0.758539	0.916028	0.897932	0.828701
**10minutes**	Adrenaline 100uM	0.692489	0.85246	0.464907	0.760478	0.513948	0.670876	0.666506	0.501271	0.789848	0.657763	0.697239	0.44235	0.652188	0.88926	0.569485	0.672965	0.527485	0.705604	0.946721	0.967394	0.695363	0.791117	0.948928	0.85297	0.849346
**20minutes**	Adrenaline 100uM	0.657282	0.862138	0.501363	0.652369	0.425649	0.558295	fainted	0.507124	fainted	0.624778	fainted	0.496692	0.690477	0.832993	0.659902	0.746696	0.556218	0.694454	1.208322	fainted	0.718645	0.822834	fainted	0.884444	0.805964

		26	27	28	29	30	31	32	33	34	35	36	37	38	39	40	41	42	43	44	45	46	47	48	49	50		
		**9_12**	**12_12**	**9_12**	**9_9**	**12_12**	**9_12**	**9_12**	**12_12**	**9_12**	**9_12**	**12_12**	**9_12**	**9_12**	**9_12**	**12_12**	**9_12**	**9_12**	**9_9**	**9_12**	**9_9**	**9_12**	**12_12**	**9_12**	**9_12**	**9_12**		
		15S062	15S063	15S066	15S071	15S072	15S073	15S075	15S076	15S077	15S078	15S079	15S081	15S083	15S085	15S086	15S087	15S088	15S089	15S090	15S094	15S096	15S098	15S102	15S104	15S106	**Average**	**SD**
**Baseline**	Adrenaline 100uM	0.621134	0.542612	0.381799	0.42352	0.454726	0.813156	0.244029	0.865517	0.43966	0.625622	0.392282	0.444764	0.453073	1.037722	1.024023	0.407368	0.765729	0.925445	0.607961	0.852563	0.822018	0.318102	0.844225	0.553122	0.750038	**0.661519**	**0.190759**
**70degree**	Adrenaline 100uM	0.640279	0.563704	0.438981	0.157284	0.452491	0.628438	0.288627	0.951188	0.369772	0.634266	0.365677	0.387788	0.343832	0.641006	0.853719	0.349006	0.786983	0.885991	0.644712	0.822483	0.788029	0.27275	0.846029	0.546991	0.695189	**0.628577**	**0.197102**
**10minutes**	Adrenaline 100uM	fainted	0.582997	0.411227	0.411613	0.268907	0.654212	0.2134	0.778274	0.413937	0.809649	0.332761	0.464108	0.866482	0.91395	1.020203	0.287932	0.85624	0.892933	0.699363	0.845331	0.868639	0.224704	0.959459	fainted	0.760684	**0.673249**	**0.215545**
**20minutes**	Adrenaline 100uM	fainted	0.570331	0.444002	0.18535	0.376359	0.412102	0.276553	0.840895	0.451788	0.79359	0.290771	fainted	0.6989	0.831976	0.875026	0.346715	0.865192	0.906034	0.71848	fainted	0.851645	0.199484	0.849807	fainted	0.654685	**0.642593**	**0.222957**

		1	2	3	4	5	6	7	8	9	10	11	12	13	14	15	16	17	18	19	20			
	**Healthy n=20**	**9_12**	**12_12**	**9_12**	**9_9**	**12_12**	**9_12**	**12_12**	**9_12**	**12_12**	**9_12**	**12_12**	**12_12**	**12_12**	**9_12**	**12_12**	**12_12**	**12_12**	**12_12**	**12_12**	**9_12**			
		HeC36-1	12C37-1	HeC38-1	9C040-1	12C41-1	HeC43-1	12C46-1	HeC47-1	C048-1	C050-1	C051-1	C052-1	C053-1	C055-1	C057-1	C058-1	C059-1	C060-1	C061-1	C062-1	**Average**	**SD**	***t*-test**
**Baseline**	Adrenaline 100uM	0.761974	0.473305	0.506677	0.504345	0.458885	0.484882	0.585582	0.488452	0.470625	0.464659	0.61892	0.429939	0.675834	0.610623	0.651512	0.572962	0.309672	0.53605	0.63296	0.906953	**0.557241**	**0.131121**	**0.005827**
**70degree**	Adrenaline 100uM	0.705793	0.417502	0.453322	0.526494	0.412419	0.357069	0.460932	0.643443	0.413427	0.418743	0.579005	0.389906	0.68229	0.529268	0.576131	0.711475	0.303575	0.654666	0.604842	0.697619	**0.526896**	**0.129246**	**0.007163**
**10minutes**	Adrenaline 100uM	0.577236	0.440056	0.519117	0.607661	0.446216	0.341682	0.390337	0.602928	0.363253	0.48952	0.535941	0.454848	0.63866	0.505325	0.652565	0.785503	0.375235	1.103886	0.664155	0.756855	**0.562549**	**0.179648**	**0.017475**
**20minutes**	Adrenaline 100uM	0.576932	0.51415	0.633898	0.547847	0.467596	0.370675	0.416147	0.619397	0.379071	0.49629	0.572353	0.489428	0.5781	0.674579	0.697558	0.796095	0.258189	0.959811	0.646144	0.774718	**0.573449**	**0.163433**	**0.088451**
