# Supplementary material for: Association of adenylate cyclase activity in vasopressor-type neurally mediated syncope based on the α2b-AR gene
Source: PLoS One. 2025 Feb 3;20(2):e0317817. doi: 10.1371/journal.pone.0317817 (PMC11790091; doi:10.1371/journal.pone.0317817)
Supplement: S3 Table — (PDF) [file pone.0317817.s003.pdf]

S3 Table. Raw data of the two genotypes and AC activity among VT-NMS patients and healthy volunteers during the HUT test.

	NMS (VT) n=19	1	2	3	4	5	6	7	8	9	10	11	12	13	14	15	16	17	18	19		
	**Glu 12/12**	S010	S015	S018	S022	S027	S028	S030	15S041	15S043	15S052	15S056	15S057	15S061	15S063	15S072	15S076	15S079	15S086	15S098	**Average**	**SD**
**Baseline**	Adrenaline 100uM	0.684176	0.532215	0.572592	0.748171	0.567394	0.742349	0.653562	0.787712	0.805212	1.087747	0.643871	0.739525	0.748586	0.542612	0.454726	0.865517	0.392282	1.024023	0.318102	**0.679493**	**0.196432**
**70degree**	Adrenaline 100uM	0.645164	0.477209	0.616481	0.742601	0.516414	0.720865	0.518816	0.752473	0.849796	1.072851	0.617435	0.758539	0.828701	0.563704	0.452491	0.951188	0.365677	0.853719	0.27275	**0.661941**	**0.204501**
**10minutes**	Adrenaline 100uM	0.692489	0.464907	0.760478	0.670876	0.501271	0.789848	0.657763	0.652188	0.88926	0.946721	0.695363	0.791117	0.849346	0.582997	0.268907	0.778274	0.332761	1.020203	0.224704	**0.661551**	**0.221065**
**20minutes**	Adrenaline 100uM	0.657282	0.501363	0.652369	0.558295	0.507124	fainted	0.624778	0.690477	0.832993	1.208322	0.718645	0.822834	0.805964	0.570331	0.376359	0.840895	0.290771	0.875026	0.199484	**0.651851**	**0.237341**

	Healthy n=12	1	2	3	4	5	6	7	8	9	10	11	12			
	**Glu 12/12**	12C37	12C41	12C46	C048	C051	C052	C053	C057	C058	C059	C060	C061	**Average**	**SD**	***t*-test**
**Baseline**	Adrenaline 100uM	0.473305	0.458885	0.585582	0.470625	0.61892	0.429939	0.675834	0.651512	0.572962	0.309672	0.53605	0.63296	**0.534687**	**0.108545**	**0.006664**
**70degree**	Adrenaline 100uM	0.417502	0.412419	0.460932	0.413427	0.579005	0.389906	0.68229	0.576131	0.711475	0.303575	0.654666	0.604842	**0.517181**	**0.133335**	**0.011913**
**10minutes**	Adrenaline 100uM	0.440056	0.446216	0.390337	0.363253	0.535941	0.454848	0.63866	0.652565	0.785503	0.375235	1.103886	0.664155	**0.570888**	**0.215627**	**0.134993**
**20minutes**	Adrenaline 100uM	0.51415	0.467596	0.416147	0.379071	0.572353	0.489428	0.5781	0.697558	0.796095	0.258189	0.959811	0.646144	**0.564553**	**0.191241**	**0.138267**

	NMS (VT) n=28	1	2	3	4	5	6	7	8	9	10	11	12	13	14	15	16	17	18	19
	**Glu 9/12**	S011	S020	S025	S033	S038	15S046	15S047	15S049	15S051	15S054	15S058	15S059	15S062	15S066	15S073	15S075	15S077	15S078	15S081
**Baseline**	Adrenaline 100uM	0.827716	0.60253	0.650868	0.704688	0.431794	0.701491	0.645822	0.532076	0.684692	0.613364	0.854632	0.902964	0.621134	0.381799	0.813156	0.244029	0.43966	0.625622	0.444764
**70degree**	Adrenaline 100uM	0.773656	0.554981	0.614422	0.66297	0.43818	0.734708	0.669328	0.468116	0.619155	0.606822	0.916028	0.897932	0.640279	0.438981	0.628438	0.288627	0.369772	0.634266	0.387788
**10minutes**	Adrenaline 100uM	0.85246	0.513948	0.666506	0.697239	0.44235	0.569485	0.672965	0.527485	0.705604	0.967394	0.948928	0.85297	fainted	0.411227	0.654212	0.2134	0.413937	0.809649	0.464108
**20minutes**	Adrenaline 100uM	0.862138	0.425649	fainted	fainted	0.496692	0.659902	0.746696	0.556218	0.694454	fainted	fainted	0.884444	fainted	0.444002	0.412102	0.276553	0.451788	0.79359	fainted

		20	21	22	23	24	25	26	27	28		
		15S083	15S085	15S087	15S088	15S090	15S096	15S102	15S104	15S106	**Average**	**SD**
**Baseline**	Adrenaline 100uM	0.453073	1.037722	0.407368	0.765729	0.607961	0.822018	0.844225	0.553122	0.750038	**0.641573**	**0.183428**
**70degree**	Adrenaline 100uM	0.343832	0.641006	0.349006	0.786983	0.644712	0.788029	0.846029	0.546991	0.695189	**0.606651**	**0.17108**
**10minutes**	Adrenaline 100uM	0.866482	0.91395	0.287932	0.85624	0.699363	0.868639	0.959459	fainted	0.760684	**0.676793**	**0.214914**
**20minutes**	Adrenaline 100uM	0.6989	0.831976	0.346715	0.865192	0.71848	0.851645	0.849807	fainted	0.654685	**0.643887**	**0.193743**

**Glu 12/12 vs. Glu 9/12**	
**t-test**	**NMS**
**0.254468**	
**0.169349**	
**0.409221**	
**0.455135**	

	Healthy n=7	1	2	3	4	5	6	7			
	**Glu 9/12**	C36	C38	C43	C47	C050	C055	C062	**Average**	**SD**	***t*-test**
**Baseline**	Adrenaline 100uM	0.761974	0.506677	0.484882	0.488452	0.464659	0.610623	0.906953	**0.60346**	**0.169905**	**0.30653**
**70degree**	Adrenaline 100uM	0.705793	0.453322	0.357069	0.643443	0.418743	0.529268	0.697619	**0.543608**	**0.140675**	**0.166464**
**10minutes**	Adrenaline 100uM	0.577236	0.519117	0.341682	0.602928	0.48952	0.505325	0.756855	**0.541809**	**0.126403**	**0.024775**
**20minutes**	Adrenaline 100uM	0.576932	0.633898	0.370675	0.619397	0.49629	0.674579	0.774718	**0.592356**	**0.129796**	**0.219061**
