# Supplementary material for: Association of adenylate cyclase activity in vasopressor-type neurally mediated syncope based on the α2b-AR gene
Source: PLoS One. 2025 Feb 3;20(2):e0317817. doi: 10.1371/journal.pone.0317817 (PMC11790091; doi:10.1371/journal.pone.0317817)
Supplement: S4 Table — (PDF) [file pone.0317817.s004.pdf]

S4 Table. Raw data on blood pressure during HUT for Glu12/12 and Glu9/12 VT-NMS patients and healthy volunteers. BP: blood pressure

				Baseline			70degrees			10minites			20minutes		
**VT-NMS**	**Number**	**Age**	**Sex**	**Systolic BP**	**Diastolic BP**	**Plus (bpm)**	**Systolic BP**	**Diastolic BP**	**Plus (bpm)**	**Systolic BP**	**Diastolic BP**	**Plus (bpm)**	**Systolic BP**	**Diastolic BP**	**Plus (bpm)**
**9/12type**	1	59	Female	111	73	48	110	83	71	96	79	72	99	78	80
**n=28**	2	20	Female	142	60	95	127	74	120	106	60	114	96	62	126
	3	67	Male	129	81	66	138	81	64	64	56	48	151	84	64
	4	67	Male	90	58	56	98	63	71	83	62	87	onset	onset	onset
	5	24	Male	108	71	56	115	116	82	101	69	73	108	69	73
	6	19	Female	121	64	89	145	84	88	102	62	101	108	64	108
	7	55	Male	115	85	81	119	93	80	117	89	87	120	93	98
	8	23	Female	112	70	56	124	84	70	108	71	74	120	86	72
	9	61	Male	138	113	57	136	120	64	154	118	72	152	122	68
	10	25	Female	112	76	66	113	71	76	107	77	100	72	36	62
	11	26	Female	108	70	62	113	93	78	102	82	91	onset	onset	onset
	12	25	Male	132	78	71	135	81	70	145	93	83	135	91	88
	13	16	Female	110	61	67	116	74	84	onset	onset	onset	onset	onset	onset
	14	17	Female	119	78	93	95	60	124	121	84	129	122	97	140
	15	69	Female	121	82	75	138	81	81	130	97	75	121	84	85
	16	19	Female	118	79	101	155	120	110	143	83	129	124	84	126
	17	20	Male	104	67	68	98	75	80	102	73	93	104	67	92
	18	20	Male	115	55	60	103	60	67	51	17	80	66	33	84
	19	21	Female	95	56	70	93	72	74	91	63	90	onset	onset	onset
	20	15	Male	75	42	73	73	43	95	onset	onset	onset	onset	onset	onset
	21	17	Female	99	69	71	85	56	105	95	69	100	97	63	98
	22	54	Male	110	76	63	114	86	69	108	82	72	125	88	74
	23	16	Female	102	51	60	106	70	79	103	65	86	94	62	112
	24	16	Male	110	61	80	135	95	90	126	90	109	131	87	121
	25	47	Female	138	102	74	136	98	77	133	107	68	126	97	79
	26	23	Female	104	69	92	99	79	120	112	76	93	125	70	116
	27	27	Female	106	73	60	113	94	114	onset	onset	onset	onset	onset	onset
	28	34	Male	94	67	60	106	80	74	104	77	74	103	73	89
		32.214286		112.07143	70.964286	70.357143	115.64286	81.642857	84.892857	108.16	76.04	88	113.59091	76.818182	93.409091
		18.671343		15.092133	14.48494	13.646908	19.433103	18.217237	18.076667	23.294635	19.329339	18.982448	21.612737	19.984843	22.374856

				Baseline			70degrees			10minites			20minutes		
**VT-NMS**	**Number**	**Age**	**Sex**	**Systolic BP**	**Diastolic BP**	**Plus (bpm)**	**Systolic BP**	**Diastolic BP**	**Plus (bpm)**	**Systolic BP**	**Diastolic BP**	**Plus (bpm)**	**Systolic BP**	**Diastolic BP**	**Plus (bpm)**
**12/12type**	1	70	Female	116	79	47	120	76	52	130	78	55	122	74	54
**n=19**	2	46	Female	112	78	67	119	86	70	105	68	73	107	71	85
	3	55	Male	108	75	44	114	80	51	96	65	54	95	70	58
	4	20	Male	127	69	68	137	79	82	116	72	98	112	73	106
	5	42	Male	115	80	55	118	80	66	113	73	73	115	70	70
	6	21	Male	107	70	55	116	73	85	onset	onset	onset	onset	onset	onset
	7	68	Male	127	76	53	113	74	56	82	59	69	91	56	75
	8	54	Female	150	78	56	152	77	57	126	83	56	132	81	58
	9	57	Male	102	72	68	118	76	81	106	76	83	91	68	73
	10	21	Male	123	68	86	141	82	81	123	72	85	129	75	71
	11	21	Female	101	61	62	127	70	68	105	77	76	101	66	79
	12	35	Female	100	62	84	115	91	112	108	73	114	95	60	125
	13	32	Female	108	79	83	125	88	92	118	87	98	112	84	107
	14	71	Male	137	98	83	126	97	98	117	87	93	105	78	101
	15	24	Female	111	80	55	104	81	61	104	73	65	110	79	77
	16	19	Male	105	62	78	101	67	99	109	71	107	103	69	118
	17	16	Male	109	63	89	106	83	103	116	80	99	123	84	102
	18	26	Female	127	79	77	148	107	83	127	83	97	149	82	89
	19	56	Female	135	88	69	158	109	78	144	98	79	131	94	83
		39.684211		116.84211	74.578947	67.315789	124.10526	82.947368	77.631579	113.61111	76.388889	81.888889	112.38889	74.111111	85.055556
		19.27881		13.853452	9.4534872	14.091182	16.192916	11.365023	18.028405	13.966931	9.0757524	18.246318	16.062887	9.2092593	20.8114

				Baseline			70degrees			10minites			20minutes		
**Healthy volunte**	**Number**	**Age**	**Sex**	**Systolic BP**	**Diastolic BP**	**Plus (bpm)**	**Systolic BP**	**Diastolic BP**	**Plus (bpm)**	**Systolic BP**	**Diastolic BP**	**Plus (bpm)**	**Systolic BP**	**Diastolic BP**	**Plus (bpm)**
**9/12type**	1	47	Female	106	71	64	132	91	74	118	81	74	104	70	73
**n=7**	2	42	Female	91	48	54	91	63	66	84	56	66	88	64	74
	3	40	Female	110	68	53	137	85	64	110	77	67	111	90	73
	4	25	Male	111	59	74	108	76	80	99	69	82	104	71	85
	5	38	Female	106	69	65	116	87	72	109	85	75	111	84	79
	6	45	Female	112	68	70	105	81	78	101	76	82	108	79	83
	7	27	Female	93	60	63	107	70	69	101	63	76	87	63	87
		37.714286		104.14286	63.285714	63.285714	113.71429	79	71.857143	103.14286	72.428571	74.571429	101.85714	74.428571	79.142857
		8.5579259		8.6299589	8.1591316	7.6966288	16.080156	9.9498744	5.9561893	10.761483	10.293317	6.3733074	10.22136	10.212038	5.9561893

				Baseline			70degrees			10minites			20minutes		
**Healthy volunte**	**Number**	**Age**	**Sex**	**Systolic BP**	**Diastolic BP**	**Plus (bpm)**	**Systolic BP**	**Diastolic BP**	**Plus (bpm)**	**Systolic BP**	**Diastolic BP**	**Plus (bpm)**	**Systolic BP**	**Diastolic BP**	**Plus (bpm)**
**12/12type**	1	42	Female	108	64	71	97	76	85	95	70	83	94	72	87
**n=12**	2	43	Male	114	75	63	111	79	78	110	78	80	105	77	88
	3	28	Male	117	76	66	133	89	65	111	94	72	114	82	75
	4	36	Female	109	67	87	117	77	90	89	63	87	88	67	91
	5	27	Female	89	57	65	90	69	79	89	63	83	89	62	94
	6	49	Female	108	64	64	109	81	73	104	68	67	97	77	77
	7	37	Male	116	83	67	108	85	65	109	76	65	106	80	65
	8	30	Female	107	56	71	89	67	73	98	58	76	97	62	78
	9	27	Female	107	71	75	116	71	64	99	61	72	110	71	87
	10	52	Female	114	78	76	123	86	83	120	84	83	111	86	88
	11	24	Male	110	60	55	108	66	65	81	47	53	95	56	61
	12	34	Male	129	76	75	146	94	79	121	74	89	124	81	91
		35.75		110.66667	68.916667	69.583333	112.25	78.333333	74.916667	102.16667	69.666667	75.833333	102.5	72.75	81.833333
		9.1465344		9.2474403	8.8570294	8.1737755	16.619403	9.0386376	8.8364774	12.561365	12.586669	10.512619	10.966892	9.3723481	10.650082

***t*-test1**	**HV9/12 vs. NMS12/12**	0.0061731	0.0054075	0.182606	0.0865242	0.2024884	0.1160129	0.0324196	0.1968229	0.0756488	0.033932	0.4721285	0.1425358
***t*-test2**	**HV9/12 vs. NMS9/12**	0.042681	0.0400561	0.0433808	0.3955168	0.3053454	0.0016759	0.2128819	0.2590586	0.0028637	0.0318399	0.3409684	0.0058725
***t*-test3**	**HV12/12 vs. NMS12/12**	0.0738186	0.0519154	0.2876938	0.0314747	0.1107977	0.2904262	0.0138089	0.0639013	0.1299172	0.0274387	0.3491545	0.2912245
***t*-test4**	**HV12/12 vs. NMS9/12**	0.3607229	0.2941474	0.4130742	0.2897836	0.2242303	0.012384	0.1585595	0.1192952	0.0086288	0.027964	0.2131305	0.0249011
***t*-test5**	**NMS9/12 vs. NMS12/12**	0.1352164	0.1531009	0.2332978	0.0562748	0.3820002	0.09184	0.1725253	0.4687478	0.1467701	0.4206746	0.2877116	0.1148982
